# Variability in Clinical Presentation of Neonatal Stroke: Report of Four Cases

**DOI:** 10.1155/2017/5215686

**Published:** 2017-01-17

**Authors:** Sonia Joseph, Dimitrios Angelis, Robert Bennett, Bhargavi Kola, Amanda Hughes

**Affiliations:** Texas Tech University Health Sciences Center, Odessa, TX, USA

## Abstract

Neonatal stroke can be a cause of long term neurodevelopmental disability, seizures, and impaired cognitive function. We present four cases of neonatal stroke, associated with different risk factors and clinical presentations. Two of these newborns were born to mothers with no prenatal care.

## 1. Introduction

Neonatal stroke is an important complication to consider when assessing a newborn as it has the potential for chronic sequelae related to neurodevelopment. Injury to cerebral tissue occurs by a disruption in arterial blood flow either from a thrombus or embolism, also known as PAIS, or from an interruption by a thrombus in a major cerebral vein, otherwise known as CSVT. Here, we present four consecutive cases of neonatal stroke over a period of two years, associated with different risk factors and different clinical presentations. Two of these babies were born to mothers with no prenatal care.

## 2. Cases

**Figure 1:**
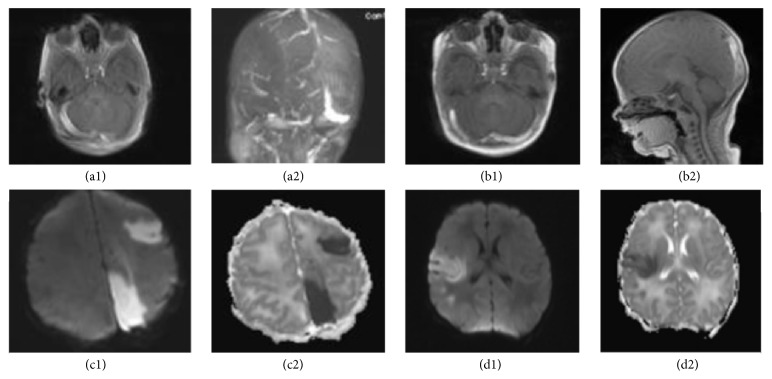
(a1, a2) Preterm neonate with severe perinatal depression born to mother with no prenatal care: the MRI and MRV images, respectively, show venous sinus thrombosis involving the entire right transverse and sigmoid sinus, most of the straight sinus, and the medial aspect of the left transverse sinus. In this case low molecular weight heparin was initiated. (b1, b2) Term neonate with sepsis, hyperthermia, and suspected episodes of seizures. On T1 images there was an abnormal signal intensity in the lower superior sagittal sinuses as well as bilateral transverse sinuses, consistent with CSVT. A small amount of intraventricular hemorrhage in the posterior body and atrium of the right lateral ventricle was also identified. Infant's coagulation work-up was negative for any major clotting disorder. (c1, c2) Diffusion weighted images (DWI) as well as ADC Map are shown for a term neonate with no identified risk factors that presented with seizures. (d1, d2) An IUGR infant admitted to the NICU due to suspected apnea and respiratory distress. An MRI without contrast demonstrated a moderately sized acute cortical infarct in the right middle cerebral artery territory, involving the right posterior frontal lobe and frontoparietal junction at the region of the motor strip.

A 1700-gram preterm female infant was born at 33 weeks' gestation to a primigravida mother with no prenatal care and unknown GBS status admitted with severe preeclampsia. Prolonged fetal heart rate decelerations resulted in an emergent C-section. At birth, the patient was hypotonic with an acidotic cord blood gas (pH = 7). She required mechanical ventilation and cardiovascular support. An echocardiogram demonstrated no intracardiac thrombi and a HUS was normal. There was an interim gradual neurologic improvement. An MRI, done at about two weeks of life, showed a thrombus in the right transverse and straight sinuses consistent with CSVT. A repeat MRI a week later was stable (Figures 1(a1) and 1(a2)). The patient was discharged home on LMWH. No coagulation defect was identified in this case. The placental pathology showed the presence of an infarct.

A 3400-gram male infant was delivered to a healthy mother with no prenatal care and an unknown GBS status at 37.4 weeks' gestation. The infant developed respiratory failure with bradycardia requiring intensive resuscitative measures (chest compression and intubation) and was admitted to the intensive care unit where he continued to have systemic compromise needing inotropes, mechanical ventilation, and blood products. On day of life one, the patient was noted to have abnormal posturing, but an EEG and HUS were negative. He developed a brief period of hyperthermia which was managed conservatively. An MRI showed abnormal signal intensity in the lower superior sagittal and bilateral transverse sinuses, indicating CSVT (Figures 1(b1) and 1(b2)). No placenta pathology was available for this patient. Following evaluation at a tertiary center, the coagulation work-up was negative.

A 2600-gram male infant was born at 39-week gestation to a healthy mother with unremarkable prenatal labs and negative GBS status. The mother presented with spontaneous ROM with clear amniotic fluid minutes before an uncomplicated vaginal delivery. Following delivery, the infant showed signs of feeding intolerance with emesis and shallow breathing. At about 10 hours of life, he proceeded to have seizure activity lasting about 10 minutes. Laboratory evaluation was significant for hypocalcemia, hypoglycemia, and thrombocytopenia. An MRI showed evidence of an acute multifocal stroke involving the left posterior, medial parietal, and left occipital lobe (Figures 1(c1) and 1(c2)). CT of the brain was negative. The patient was followed conservatively. Placenta was not evaluated in this case. The KB test was positive revealing a mild to moderate fetal-maternal hemorrhage.

A 1970-gram male infant, with IUGR, was born at 38 weeks' gestation to a GBS negative mother with a history of diabetes mellitus type 2 and smoking. The mother developed preeclampsia. Shortly after birth, he experienced episodes of desaturation. CXR was within normal limits. Subsequently, he developed two episodes of suspected central apnea, requiring stimulation. CT scan and HUS were negative. However, an MRI without contrast demonstrated an acute cortical infarct in the right MCA, involving the posterior frontal lobe and frontoparietal junction (Figures 1(d1) and 1(d2)). Further neurological and hematological evaluation failed to demonstrate any significant defect. Conservative management was followed for this patient. Placental pathology was consistent with acute infection as well as a small placental infarct.

## 3. Discussion

Perinatal stroke is defined as a cerebrovascular accident occurring between 20 weeks' gestation and up to 28 days after birth in the newborn period, causing neurological damage in the territory of the affected vessel and persisting for greater than 24 hours [1]. The classification includes perinatal arterial ischemic strokes (PAIS), cerebral sinus venous thrombosis (CSVT), or hemorrhagic strokes. PAIS is reported in 1 in 2300–4000 deliveries as compared to the rarer CSVT which occurs between 1 and 2.69 per 100,000 births. CSVT occurs when venous blood flow in a major sinus becomes disrupted. About 60% of reported CSVT cases involve a parenchymal infarction [2].

Our cases demonstrate the variability of the clinical presentation of neonatal stroke. Nonspecific neurologic signs and symptoms (such as respiratory failure, temperature instability) along with the typical neurologic symptoms (seizures, abnormal posturing) could denote a disruption in supply or demand of oxygen in the vulnerable neonatal brain. Seizures are the presenting symptom for newborns with stroke in the first month of life, accounting for 70–90% of these cases [3]. Only 50% of newborns present with typical focal seizures and up to 20% of these events are subtle and difficult to recognize [4]. A high index of suspicion is required for newborns born to mothers with specific risk factors, presenting with subtle neurologic signs.

PNC is a well-established and effective way to monitor maternal and fetal health and identify women at risk for unfavorable birth outcomes [5]. Early and continuous PNC helps the delivery of an array of medical, nutritional, and educational interventions at different stages of pregnancy [6]. Despite recommendations, a proportion of women continue to receive insufficient or no PNC [6]. Absence of prenatal has many reasons including socioeconomic variable and racial differences. A retrospective cohort study found that about one in five women had less than adequate PNC with striking racial differences [7]. In our cases two of the four mothers, all with CSVT, had no prenatal care. Due to the rarity of neonatal stroke and the small number of cases it would be difficult to speculate any association between absence of prenatal care, socioeconomic factors, and neonatal stroke and further studies are required.

In Table 1 we summarize the known risk factors associated with neonatal stroke, in these cases. Preeclampsia, the presenting symptom in 2 of our cases, is a hypercoagulable status in the fetus and is associated with decreased placental blood flow. In a case-control study, which included 40 newborns with stroke it was found that preeclampsia, chorioamnionitis, PROM, and infertility were independently associated with PAIS [8]. The investigators concluded that perinatal risk factors could act synergistically. PNC was not adequately assessed and socioeconomic factors and social habits were not reported. Only 3 of these 40 cases had a documented placental pathology report. In our study 2 patients had a placental infarct seen at the pathology report. Placental infarction and inflammatory changes have been reported in patients with cerebral palsy, but not in patients with stroke and hence it is difficult to interpret their significance [9]. Similar associations regarding the presence or absence of specific risk factors have been described for CSVT [10]. Interestingly, primiparity is an important risk factor for PAIS in term gestations, but this association appears to be weak in preterm infants and in those with CSVT [11, 12].

MRI is considered the study of choice for identifying and diagnosing perinatal stroke in term babies. Perinatal stroke occurs mainly in full-term infants and in lower incidence in preterm infants [13]. PAIS is more common on the left side, in both term and preterm newborns, and usually follows the distribution of the MCA. In preterm babies due to the involvement of several MCA lenticulostriate branches, serial HUS might be more sensitive than early MRI for identification of a type of stroke that is common in this age group. Plaisier et al. following a specific protocol of serial HUS found that MRI was superior in identifying cerebellar hemorrhage but serial ultrasonography in detection of IVH, perforator stroke, and CSVT [14]. In preterm newborns, CSVT typically should be considered when there is bilateral white matter involvement, often associated with an intraventricular hemorrhage. Doppler flow ultrasonography, if available, could help in the diagnosis. In another study newborns had a power Doppler HUS, which detected about 50% of the instances of CSVT [15]. In our case, serial HUS failed to identify the CSVT in this preterm infant. This newborn did not have white matter abnormalities or hemorrhage that could raise the suspicion of an underlying venous thrombosis and Doppler ultrasonography was not performed routinely in the particular institution. For cases like this an MRI combined with an MRV is necessary for confirmation of the diagnosis of CSVT.

## 4. Conclusion

This was a descriptive study on the etiology, clinical presentation, management, and necessary work-up of neonatal stroke in a regional neonatal population with limited prenatal care. Our case series illustrated four different presentations of strokes in the perinatal period. While the management of most perinatal strokes is supportive, early recognition allows for the focus to be placed on treatment of underlying conditions and preventing further injury.

## Figures and Tables

**Table 1:** 

	Case 1	Case 2	Case 3	Case 4
Gestational age	33 weeks	37.4 weeks	39 weeks	38 weeks

Presence of prenatal care	None	None	Yes	Yes

Risk factors associated with stroke	Preeclampsia, primiparity	Resuscitation at birth	None	Preeclampsia, IUGR, smoking, obesity H/o diabetes mellitus type 2 (normal GTT)

Presenting symptoms	Hypotonia, respiratory distress	Central apnea, hyperthermia, respiratory distress	Seizures	Central apnea, respiratory distress

APGAR score at 1, 5, 10 min	1, 5, 8	8, 1, 7	8, 9, 9	8, 9, 9

Clinical course	Receiving prolonged mechanical ventilation Requiring inotropes and blood products Clinical recovery	Requiring prolonged mechanical ventilation, Receiving inotropes, transfusions of FFP, packed RBCs Clinical recovery	Seizure activity controlled with AED Requiring high GIR and calcium gluconate supplements Clinical recovery	Mild oxygen requirements Clinical recovery

Placental pathology	Placental infarct	Not performed	Not performed + KB test	Acute funisitis, acute chorioamnionitis, placental infarct

Coagulation work-up	Negative	Negative	Negative	Negative

Follow-up of the patient	Anticoagulation	Conservative management	Conservative management	Conservative management

## Abbreviations

AED: Antiepileptic drugs
aPTT: Activated partial thromboplastin time
CSVT: Cerebral sinus venous thrombosis
CT: Computed tomography
CXR: Chest radiograph
EEG: Electroencephalogram
ECG: Electrocardiogram
FFP: Fresh Frozen Plasma
GBS: Group B* Streptococcus*
GIR: Glucose Infusion Rate
GTT: Glucose tolerance test
HUS: Head ultrasound
IUGR: Intrauterine growth restriction
IVH: Intraventricular hemorrhage
KB: Kleihauer-Betke test
LMWH: Low molecular weight heparin
MCA: Middle cerebral artery
MRI: Magnetic resonance imaging
MRV: Magnetic resonance venography
NICU: Neonatal intensive care unit
PAIS: Perinatal arterial ischemic stroke
PNC: Prenatal care
PROM: Prolonged rupture of membranes
RBC: Red blood cells.
